# Delivering Cardiac Rehabilitation Exercise Virtually Using a Digital Health Platform (ECME-CR): Protocol for a Pilot Trial

**DOI:** 10.2196/31855

**Published:** 2021-10-07

**Authors:** Oonagh M Giggins, Julie Doyle, Suzanne Smith, Orla Moran, Shane Gavin, Nisanth Sojan, Gordon Boyle

**Affiliations:** 1 NetwellCASALA Dundalk Institute of Technology Dundalk Ireland

**Keywords:** cardiac rehabilitation, exercise, cardiovascular disease, virtual rehabilitation, digital health, self-management, pilot study, platform, feasibility

## Abstract

**Background:**

Exercise-based cardiac rehabilitation is recognized as a core component of cardiovascular disease management and has been shown to reduce all-cause and cardiovascular mortality and reduce the risk of hospital readmission following a cardiac event. However, despite this, the uptake of and long-term adherence to cardiac rehabilitation exercise is poor. Delivering cardiac rehabilitation exercise virtually (ie, allowing patients to participate from their own homes) may be an alternative approach that could enhance uptake and increase adherence.

**Objective:**

The aim of this study is to assess the feasibility of delivering a virtual cardiac rehabilitation exercise program supported by the Eastern Corridor Medical Engineering – Cardiac Rehabilitation (ECME-CR) platform.

**Methods:**

A convenience sample (n=20) of participants eligible to participate in community-based cardiac rehabilitation exercise will be recruited. Participants will be randomized to one of two study groups. Both study groups will perform the same exercise program, consisting of twice-weekly sessions of 60 minutes each, over an 8-week intervention period. Participants in the intervention group will partake in virtually delivered cardiac rehabilitation exercise classes in their own home. The virtual exercise classes will be delivered to participants using a videoconferencing platform. Participants in the control group will attend the research center for their cardiac rehabilitation exercise classes. Intervention group participants will receive the ECME-CR digital health platform for monitoring during the class and during the intervention period. Outcomes will be assessed at baseline and following the 8-week intervention period. The primary outcome will be exercise capacity as assessed using the 6-minute walk test. Other outcome measures will include heart rate, blood pressure, weight, percentage body fat, muscle strength, and self-reported quality of life. Semistructured interviews will also be conducted with a subset of participants to explore their experiences of using the digital platform.

**Results:**

Participant recruitment and data collection will begin in July 2021, and it is anticipated that the study results will be available for dissemination in spring 2022.

**Conclusions:**

This pilot trial will inform the design of a randomized controlled trial that will assess the clinical effectiveness of the ECME-CR digital health platform.

**International Registered Report Identifier (IRRID):**

PRR1-10.2196/31855

## Introduction

Cardiovascular disease (CVD) remains the number one cause of death globally with age, high cholesterol, high blood pressure, smoking, and diabetes among the main risk factors. Although mortality from CVD has fallen over recent decades, it still results in 3.9 million deaths per year in Europe and costs the European economy €210 billion (US $248 billion) per year [1]. Due to improved survival rates, large numbers of people are living with chronic CVD. The effective management of those with CVD presents a significant challenge to health care systems globally.

Cardiac rehabilitation (CR) is recognized as a core component of CVD management, aiding in the recovery from an acute cardiac event and helping to prevent further illness and mortality. CR is generally prescribed to patients following a coronary angioplasty or coronary artery bypass graft, as well as to those with chronic heart failure. Cardiac rehabilitation typically includes nutritional counselling, risk factor management, psychosocial interventions, and lifestyle modification and education programs, as well as physical activity and exercise training. CR normally includes four phases of varying time frames: phase I (in-hospital patient period), phase II (postdischarge pre-exercise period), phase III (exercise and education program), and phase IV (maintenance). Phases III and IV are usually delivered in hospital outpatient departments or community centers. Active participation in the exercise training component of phases III and IV CR has been shown to be an effective tool in reducing all-cause and cardiovascular mortality [2,3], reduce the risk of hospital readmission [4], and have positive effects on cardiovascular risk factors, aerobic capacity, anxiety, and depression [4-6].

However, many patients do not receive appropriate CR. The COVID-19 pandemic severely impacted the delivery of CR services, with CR among the first clinical services to close at the onset of the pandemic [7]. However, even before the COVID-19 pandemic, CR was a significantly underused resource, with participation rates of around 40% being reported in recent years [8]. Multiple barriers to participation exist, such as distance to the CR center, lack of time, and the cost of rehabilitation [9]. As a result, calls have been made for alternatives to center-based rehabilitation programs [10]. Home-based CR has been advocated for many years and has been shown to be as effective as hospital-based CR in improving functional capacity [11]. The COVID-19 pandemic resulted in many CR services delivering classes virtually to patients via videoconferencing platforms [12]. However, with home-based and virtually delivered rehabilitation, clinicians have little insight as to how the patient is exercising and whether they are performing the exercises correctly and at a safe intensity. New innovative solutions are consequently required to support people with CVD to undertake their CR exercise safely and effectively at home.

**Figure 1:**
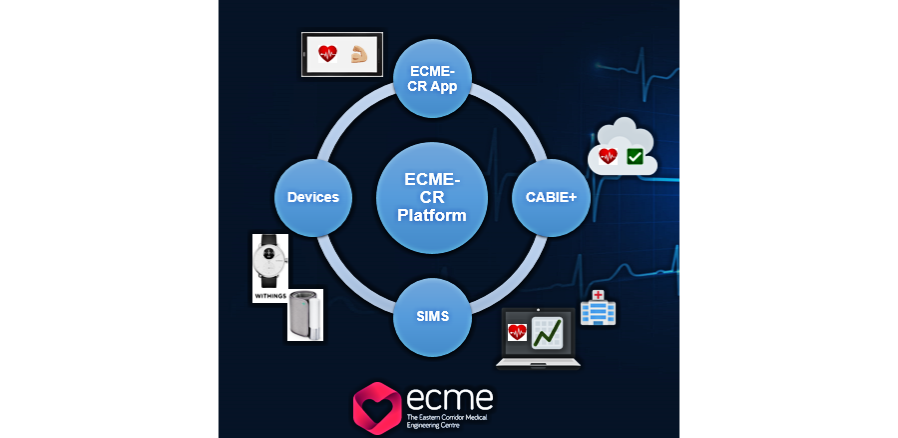
Overview of the ECME-CR platform. ECME-CR: Eastern Corridor Medical Engineering – Cardiac Rehabilitation.

In this paper, we present Eastern Corridor Medical Engineering – Cardiac Rehabilitation (ECME-CR), an interactive digital health platform (Figure 1) for cardiac rehabilitation developed by the Eastern Corridor Medical Engineering (ECME) research team at NetwellCASALA, Dundalk Institute of Technology. The platform has been designed to support the virtual delivery of CR exercise. The platform facilitates self-monitoring during the structured CR exercise class and during the intervention period.

**Figure 2:**
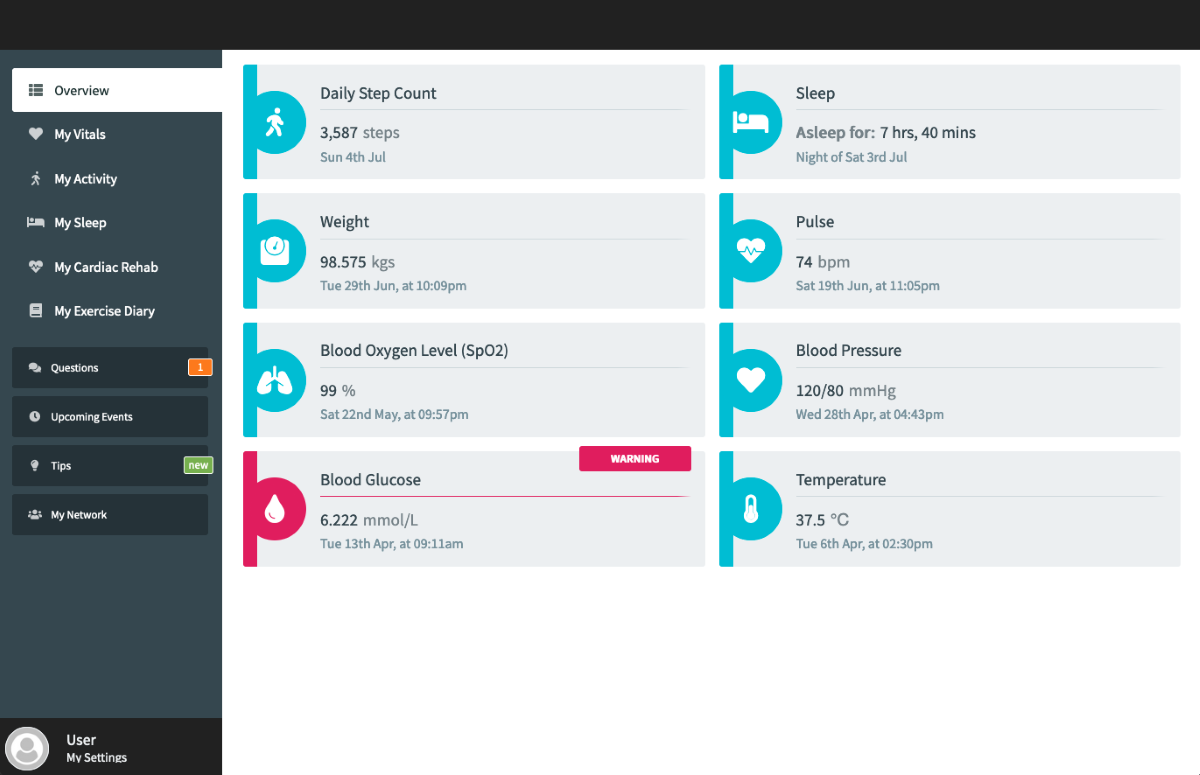
ECME-CR app dashboard. ECME-CR: Eastern Corridor Medical Engineering – Cardiac Rehabilitation.

**Figure 3:**
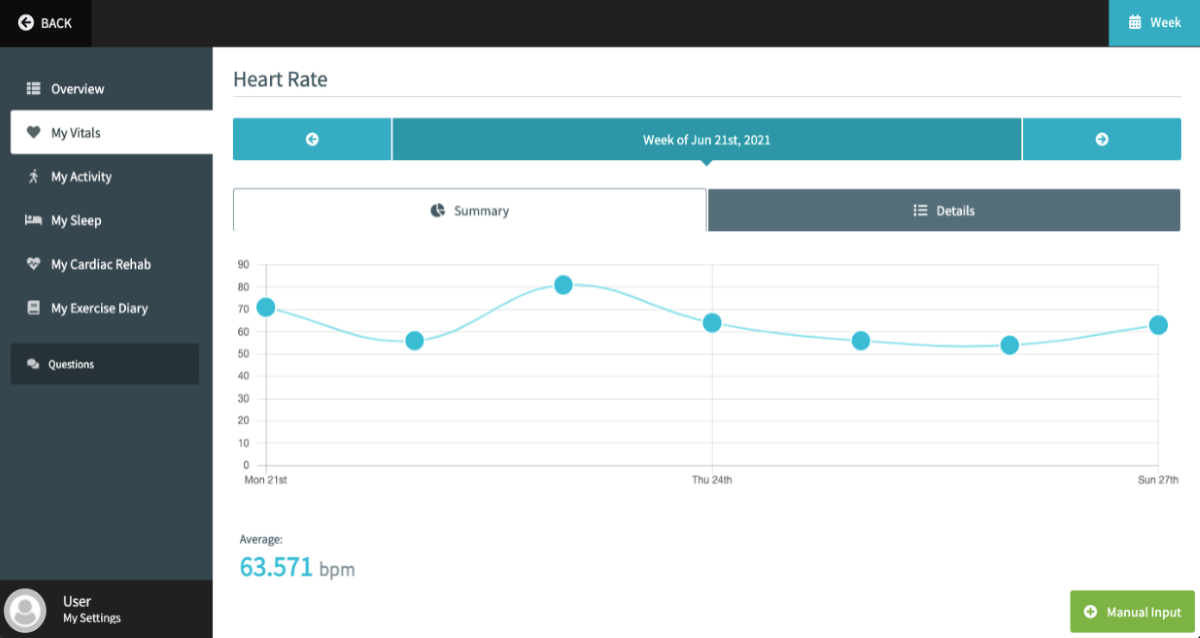
ECME-CR app: view daily average heart rate. ECME-CR: Eastern Corridor Medical Engineering – Cardiac Rehabilitation.

**Figure 4:**
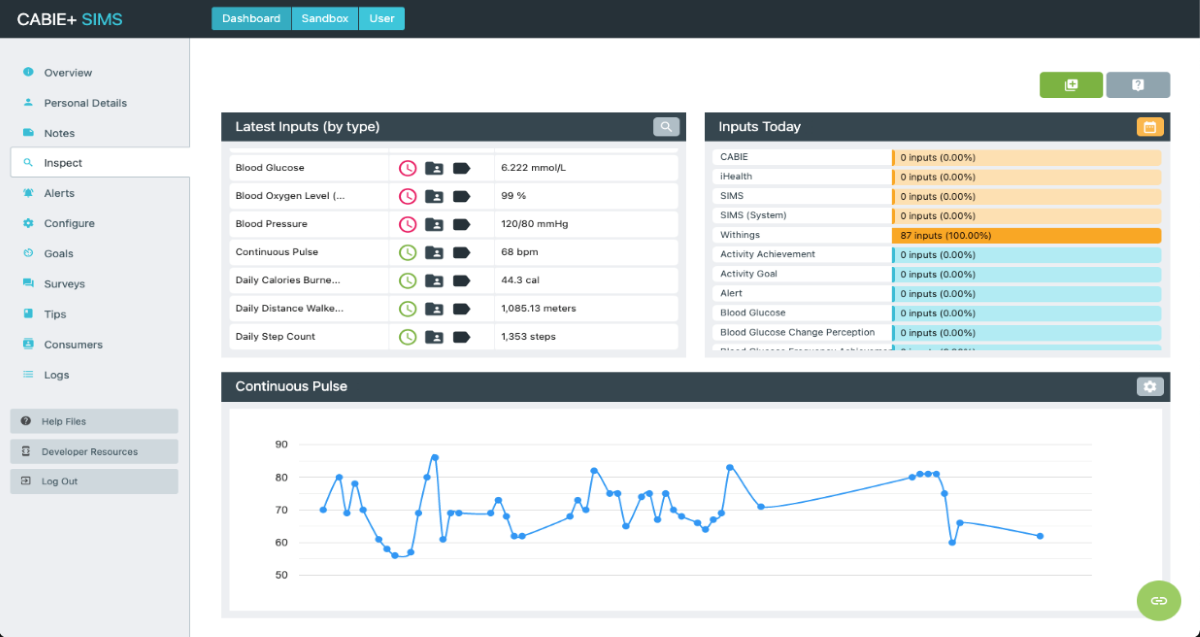
SIMS interface showing inspection of daily heart rate data.

The ECME-CR platform consists of the following components:

- The ECME-CR app, a web-based app for guidance, monitoring, and support during the CR exercise class. Participants will interact with the app via a tablet device that will be given to them for the duration of the study. Outside of the CR exercise sessions, participants can also use the app to view data (blood pressure, heart rate, activity, and sleep) from digital devices (Figure 2, Figure 3). Preinstalled educational content relating to exercise and CVD will be accessible to participants on the app at any time during the intervention period (Multimedia Appendix 1). The design of the app is based on learnings from previous research, including interviews and co-design sessions involving older adults with cardiac conditions [13,14]. Further details of how the ECME-CR app will be used by participants during the exercise classes are outlined below, in the section “Virtual Cardiac Rehabilitation.”
- Two off-the-shelf consumer devices, the Withings ScanWatch and the Withings BPM Connect, which are integrated with the platform and used to collect health and well-being data during the virtually delivered CR exercise class as well as during the intervention period. The Withings ScanWatch is a high-end smart watch with an embedded photoplethysmogram sensor for measuring heart rate and oxygen saturation, a triaxial accelerometer for monitoring activity, and electrodes for electrogram recording. The BPM Connect is a clinically validated blood pressure and heart rate monitor. Both devices connect to the Withings Health Mate app via Bluetooth.
- CABIE+, the data collection and aggregation system, which organizes and stores the data acquired from the ECME-CR app and integrated devices.
- SIMS, the information management system, which allows the exercise instructor/research team to view, analyze, and interpret the data collected from the app and the devices in near real time for individual participants (Figure 4).
  The CABIE+ and SIMS components of the platform have been described in detail elsewhere [13].
  The aim of this pilot trial is to assess the efficacy of a virtually delivered CR exercise program supported by the ECME-CR platform. The effectiveness of the CR intervention will be compared with a control group of individuals who will receive a traditional center-based CR exercise intervention. The primary outcome will be cardiopulmonary exercise capacity as assessed using the 6-minute walk test (6MWT) [15]. The effectiveness of the digital intervention will also be assessed in terms of muscle strength and health-related outcome measures taken before and after the intervention period. The pilot trial will also examine the acceptability and safety of delivering CR exercise classes virtually and will evaluate the usability and acceptability of the digital health platform. Findings from the pilot will inform the feasibility assessment for conducting a full randomized controlled trial.

## Methods

### Study Design

This is a randomized controlled pilot feasibility trial that will examine the efficacy of virtual CR exercise supported by the ECME-CR platform. This study takes an exploratory mixed methods approach and—in addition to the quantitative data generated through the feasibility trial—will generate qualitative data to understand the experiences of participating in virtual CR exercise classes and of using the digital platform.

### Ethical Considerations

All study materials and procedures have been reviewed and approved by the Human Research Ethics Committee of Dundalk Institute of Technology. Written informed consent will be obtained from all study participants prior to their participation in the study.

### Participants

**Figure 5:**
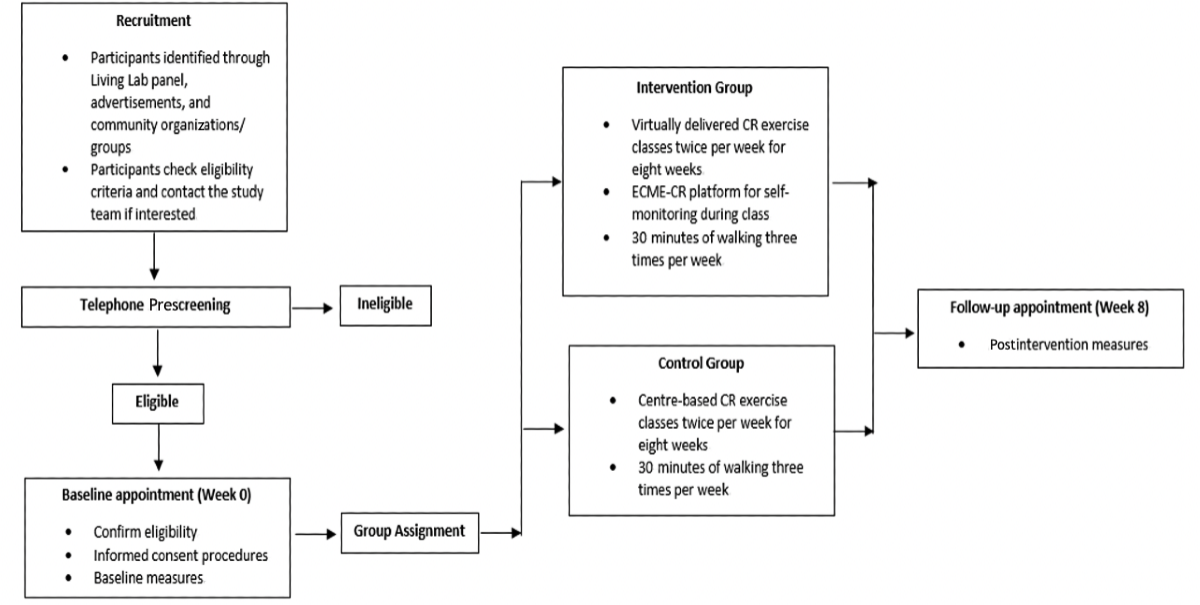
Participant flow through the study. CR: cardiac rehabilitation.

A convenience sample (n=20) of participants eligible to participate in community-based phase IV CR will be recruited. Sample size calculations were not conducted as this is a pilot trial. The sample size of 20 was selected based on the number of participants that could be conveniently recruited and tested within the pilot study time frame. Participant flow through the study is outlined in Figure 5.

Participants will be recruited from a living lab panel within Dundalk Institute of Technology, and through advertisement in local general practitioner practices, health clinics, and local media, and through community organizations and groups. Potential participants will also be informed of the study while attending outpatient CR sessions in local hospitals. Participants will be asked to contact the study team if interested in taking part. Those that make contact will initially be screened for their eligibility to take part by a member of the research team, over the phone, using the study eligibility criteria (Textbox 1).

Those that are deemed eligible and are willing to participate will then be invited to make an appointment to attend the research center for baseline testing. At this baseline appointment, the researcher will confirm the participant’s eligibility and participants will sign the informed consent document should they agree to proceed with the study.

**Inclusion and exclusion criteria.**

**Inclusion criteria**

Participants will be included if they meet the following criteria:

- Men/women with documented cardiovascular disease eligible to participate in a community-based cardiac rehabilitation program (Phase IV cardiac rehabilitation)
- Aged 40-80 years
- Medically stable with regard to symptoms and no change in pharmacotherapy in the previous 4 weeks
- Clinical approval from their treating physician to enroll in the cardiac rehabilitation program.

**Exclusion criteria**

Participants will be excluded if any of the following exclusion criteria apply to them:

- Live in a nursing home or other long-term care facility
- Have any contraindications to exercise (adapted from the American College of Sports Medicine’s Guidelines for Exercise Testing and Prescription [16]):
  Unstable angina
  Uncontrolled hypertension (ie, resting systolic blood pressure >180 mm Hg or resting diastolic blood pressure >110 mm Hg)
  Orthostatic blood pressure drop of >20 mm Hg with symptoms
  Significant aortic stenosis (aortic valve area <1.0 cm2)
  Acute systemic illness or fever
  Uncontrolled atrial or ventricular arrhythmias
  Uncontrolled sinus tachycardia (heart rate >120 beats per minute)
  Acute pericarditis or myocarditis
  Uncompensated heart failure
  Third degree (complete) atrioventricular block without pacemaker
  Recent embolism
  Acute thrombophlebitis
  Resting ST segment displacement (>2 mm)
  Uncontrolled diabetes mellitus
  Severe orthopedic conditions that would prohibit exercise
  Other metabolic conditions, such as acute thyroiditis, hypokalemia, hyperkalemia, or hypovolemia (until adequately treated)

### Group Assignment

After providing informed consent, participants will be randomly assigned to one of the two study groups. Intervention group participants will follow a virtually delivered CR exercise program in their own home and will receive support via the ECME-CR app. Control group participants will receive usual care only (ie, traditional center-based rehabilitation). Randomization schedules will be generated using a computerized random number generator. To minimize selection bias, an independent researcher will oversee the randomization process. Given the nature of the intervention, it will not be possible to blind the participant nor the outcome assessors to group allocation.

### Cardiac Rehabilitation Exercise Program

#### Overview

Both study groups will perform the same exercise rehabilitation program over an 8-week intervention period. The exercise program will be delivered by a physiotherapist and an exercise therapist with experience in CR exercise, and will consist of 60 minutes of exercise per session with two sessions per week. Each 60-minute session will consist of a 15-minute warm-up, 30 minutes of circuit style aerobic and strength exercises, and a 10-minute cooldown (Multimedia Appendix 2).

Exercise intensity will be assessed during the exercise class by self-report using the Borg scale of perceived exertion [17] and by measurement of heart rate. The level of perceived exertion on the Borg scale should commence at “very light” and gradually progress toward “somewhat hard” during the session [18]. For heart rate, the target range during exercise is 40%-70% of heart rate reserve [18], which will be calculated using the age-adjusted Karvonen formula [19]. The ScanWatch will be used during the classes to measure heart rate.

At the beginning of each class, participants will be prompted to activate the workout mode on their ScanWatch. The ScanWatch ordinarily takes a measurement of heart rate approximately every 10 minutes; however, when the workout mode is activated, the sampling frequency increases to approximately every three seconds. This high-resolution data will not be available in real time during the class; however, it will be available for review by a member of the research team following the class to assess if the participant was exercising within a safe and effective heart rate. Individualized feedback and tailoring of exercises will be provided to participants based on the postclass review of heart rate data.

During the intervention period, participants in both groups will also be encouraged to undertake additional aerobic exercise (ie, walking) 3 times per week. Participants will be encouraged to progress their activity gradually over the intervention period by first increasing the duration of the activity (ie, covering a greater distance) and then increasing the intensity gradually (ie, increasing the speed of walking). Participants in both groups will self-report the activities undertaken and this data will be included in the analysis. Participants will be guided to include a warm-up and cooldown as part of their activity.

#### Intervention Group

The intervention group will undertake the exercise program in their own home, joining virtual CR exercise classes. The virtual classes will be delivered using the videoconferencing platform Zoom (Zoom Video Communications Inc), enabling two-way interaction and communication between the instructors and participants. The instructors will guide the participants through the exercise class and will deliver real-time feedback, encouragement, and modifications.

Participants will be provided with a tablet device (10.2-inch iPad Wi-Fi 32GB, 8th generation; Apple Inc) preloaded with the ECME-CR app, the Withings devices, and a set of free weights that will be used during the exercise class. A second tablet device will be provided to participants if they do not have access to their own tablet/PC to join the Zoom CR exercise classes. Intervention group participants will be required to have an established broadband connection in their home; however, if this is not the case, a mobile Wi-Fi device will be given to them for the duration of the study. Participants will receive an equipment familiarization session either in person in the research center or during a home visit, which will include how to operate the tablet and ECME-CR app, how to use the monitoring equipment, and how to record measurements. An equipment manual with written and pictorial instructions will also be supplied.

**Figure 6:**
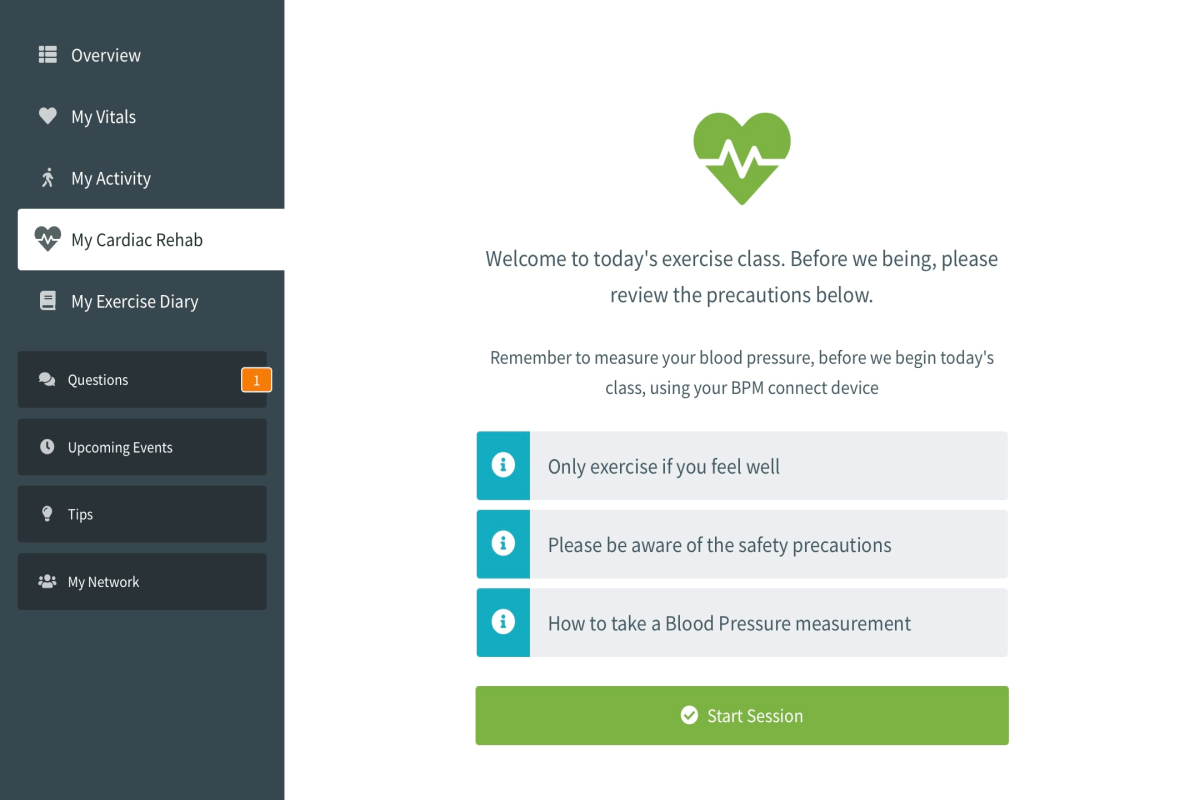
ECME-CR app during the virtual CR exercise class. CR: cardiac rehabilitation; ECME-CR: Eastern Corridor Medical Engineering – Cardiac Rehabilitation.

**Figure 7:**
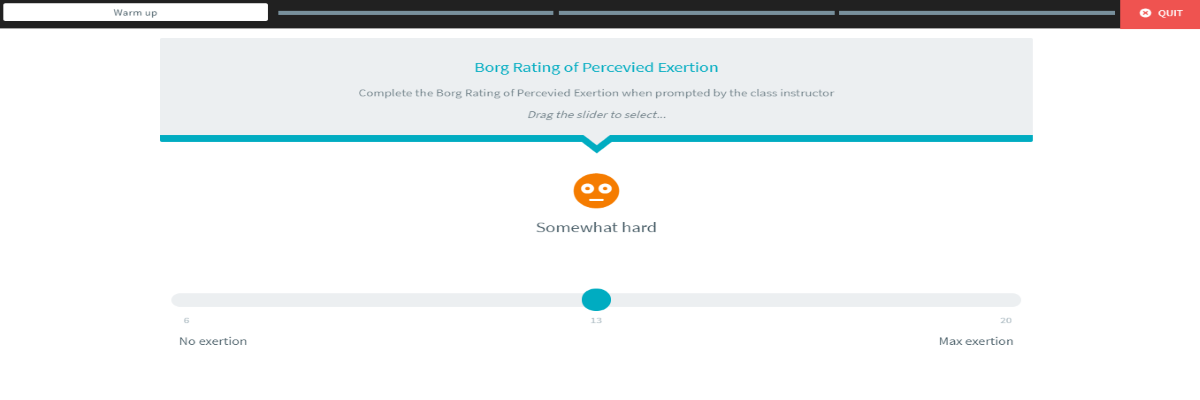
ECME-CR app: recording exertion levels on the Borg scale. ECME-CR: Eastern Corridor Medical Engineering – Cardiac Rehabilitation.

The ECME-CR app will offer guidance to participants during the exercise class (see Figure 6 for examples of the guidance that will be provided). Participants will also use the ECME-CR app to record exertion levels on the Borg scale as the class progresses (Figure 7). In addition, 5 minutes before each class begins, participants will measure their heart rate and blood pressure at rest using the BPM Connect device. These measurements will automatically synchronize with the ECME-CR app and this data will be available for review by the class instructors on SIMS before the class begins, ensuring it is safe for the participant to exercise. Participants in the intervention group will also use the ECME-CR app to self-report any additional aerobic exercise activities (type, duration, and intensity) undertaken during the intervention period.

#### Control Group

The control group will attend the research center to undertake their rehabilitation exercise classes. Each participant’s heart rate and blood pressure will be measured at rest using the Withings BPM Connect before beginning the exercise class and again following the cooldown period. Participants will be provided with a ScanWatch to wear for the duration of the class for continuous heart rate measurement. Exertion levels will be monitored at regular intervals and manually recorded by a member of the research team. Participants in the control group will record any additional aerobic exercise activities (type, duration, and intensity) undertaken outside of the classes during the intervention period in a paper diary provided to them.

### Outcome Measures

Outcome measures will be assessed at baseline (week 0) and repeated following the intervention period (week 8).

#### Primary Outcome

The primary outcome will be cardiopulmonary exercise capacity as assessed using the 6MWT [15]. The 6MWT will be performed as per the European Respiratory Society/American Thoracic Society’s guidelines [20]. Briefly, the 6MWT will be conducted in a quiet indoor corridor that is flat and straight, with a hard surface. The walking course will be 30 meters in length. The starting line—which marks the beginning and end of each 60-meter walking lap—will be marked with brightly colored tape, while the turnaround point will be marked with a cone. The 6MWT distance will be recorded to the nearest meter. The test will be performed twice to account for a learning effect [20] and the longer distance will be used in the analysis. Heart rate will be recorded during the 6MWT using the Withings ScanWatch and the data will be synchronized with the Withings Health Mate app.

#### Secondary Outcomes

Other outcomes will include measurement of strength via measurement of grip strength. Maximum grip strength for each hand will be measured in kilograms using a digital handheld isokinetic dynamometer (Takei 5401, Takei Scientific Instruments Co Ltd). A total of three maximum voluntary grip squeeze contractions will be taken for each hand, alternating between the right and left hand each time. Participants will be assessed in a standardized position and standardized encouragement will be delivered. The best measurement for each hand will be used in the analysis.

Self-reported quality of life will be assessed using a paper-based version of the 12-Item Short Form Survey [21]. Physical health–related outcome measures will be assessed, including measurement of heart rate at rest, blood pressure, weight, and BMI, as well as percentage body fat as measured using the Marsden MBF-6010 Body Composition Scale bioimpedance scale (Marsden Weighing Group) and waist circumference. Heart rate and Borg scale data collected during the CR exercise classes will also be used in the analysis. Participant adherence to the exercise program, engagement with the ECME-CR app and digital devices, and trends in daily physical activity, heart rate, and blood pressure over the intervention period will also be analyzed.

Following the intervention, interviews will be conducted to explore the experience of participating in the virtual CR exercise classes and using the ECME-CR platform. A subset of participants who were assigned to the intervention group will be invited to participate in a semistructured interview conducted either face-to-face, via Zoom, or by telephone. Semistructured interviews will also be conducted with participants in the control group to explore their perceptions of virtual CR exercise classes and what barriers and facilitators may exist to participating in this type of program. An interview schedule will be developed to guide the interviews, which will take approximately 30 minutes. Interviews will be audio recorded and subsequently transcribed verbatim.

### Data Analysis

Quantitative data collected at week 0 and week 8 will be collated using Microsoft Excel (Microsoft Corp) and the statistical software package SPSS (version 26; IBM Corp) will be used to analyze the data. Descriptive statistics will be used to describe the data. Data will be presented as frequencies, means, standard deviations, and percentages. Inferential statistical tests (*t* test) will be applied to determine whether there are differences within and between groups following the intervention. The Shapiro-Wilk test will be applied to assess normality and a significance level of *P*<.05 will be applied. Data in SIMS, collected from the app and from the digital devices, will be descriptively analyzed.

The interview transcripts will be coanalyzed by two researchers using NVivo (QSR International) following the thematic analysis process suggested by Braun and Clarke [22].

## Results

Participant recruitment and data collection began in July 2021. Dissemination of study results in peer-reviewed journals is expected in spring 2022.

## Discussion

### Principal Findings

The benefits of participating in CR exercise are well documented, with positive effects for both the individual and the health care system. Despite this, participation rates are low and adherence to CR exercise programs is poor, with many barriers being reported, such as transport difficulties, financial cost, and the lack of program availability [10]. The COVID-19 pandemic has also had an impact on the delivery of CR, with many CR exercise classes being delivered online [12]. Consequently, now more than ever, it is important to develop solutions to support people with CVD to undertake their CR exercise program virtually at home.

In this paper, we present the protocol of a pilot trial that will examine the feasibility of delivering a virtual CR exercise program supported by the ECME-CR platform, a custom-developed digital platform for self-monitoring during CR exercise. We hypothesize that the virtually delivered CR exercise program will result in similar outcomes and will not be inferior to the center-based program. We anticipate that this study will also demonstrate that virtually delivered CR exercise is a safe and acceptable alternative for those who cannot attend or complete traditional center-based CR exercise classes. The sample size in this study will limit the statistical power of the results; nevertheless, as this is a pilot trial, the proposed N is appropriate for meeting the study objectives.

### Conclusions

The outcomes of this pilot trial will inform the design of a larger randomized controlled trial that will assess the clinical effectiveness of the ECME-CR digital health platform.

## Appendix

Multimedia Appendix 1 Educational content.

Multimedia Appendix 2 Exercise program.

## Abbreviations

CR: cardiac rehabilitation
CVD: cardiovascular disease
ECME: Eastern Corridor Medical Engineering
6MWT: 6-minute walk test

## References

[1] Wilkins E, Wilson L, Wickramasinghe K, Bhatnagar P, Leal J, Luengo-Fernandez R, Burns R, Rayner M, Townsend N (2017). European Cardiovascular Disease Statistics 2017. European Heart Network.

[2] Heron N, Kee F, Donnelly M, Cardwell C, Tully MA, Cupples ME (2016). Behaviour change techniques in home-based cardiac rehabilitation: a systematic review. Br J Gen Pract.

[3] Taylor RS, Brown A, Ebrahim S, Jolliffe J, Noorani H, Rees K, Skidmore B, Stone JA, Thompson DR, Oldridge N (2004). Exercise-based rehabilitation for patients with coronary heart disease: systematic review and meta-analysis of randomized controlled trials. Am J Med.

[4] Anderson L, Oldridge N, Thompson DR, Zwisler A, Rees K, Martin N, Taylor RS (2016). Exercise-Based Cardiac Rehabilitation for Coronary Heart Disease: Cochrane Systematic Review and Meta-Analysis. J Am Coll Cardiol.

[5] Lawler PR, Filion KB, Eisenberg MJ (2011). Efficacy of exercise-based cardiac rehabilitation post-myocardial infarction: a systematic review and meta-analysis of randomized controlled trials. Am Heart J.

[6] Verschueren S, Eskes AM, Maaskant JM, Roest AM, Latour CHM, Op Reimer WS (2018). The effect of exercise therapy on depressive and anxious symptoms in patients with ischemic heart disease: A systematic review. J Psychosom Res.

[7] Vigorito C, Faggiano P, Mureddu GF (2020). COVID-19 pandemic: what consequences for cardiac rehabilitation?. Monaldi Arch Chest Dis.

[8] Kotseva K, Wood D, De Bacquer D, EUROASPIRE investigators (2018). Determinants of participation and risk factor control according to attendance in cardiac rehabilitation programmes in coronary patients in Europe: EUROASPIRE IV survey. Eur J Prev Cardiol.

[9] De Vos C, Li X, Van Vlaenderen I, Saka O, Dendale P, Eyssen M, Paulus D (2013). Participating or not in a cardiac rehabilitation programme: factors influencing a patient's decision. Eur J Prev Cardiol.

[10] Bakhshayeh S, Sarbaz M, Kimiafar K, Vakilian F, Eslami S (2021). Barriers to participation in center-based cardiac rehabilitation programs and patients' attitude toward home-based cardiac rehabilitation programs. Physiother Theory Pract.

[11] Imran HM, Baig M, Erqou S, Taveira TH, Shah NR, Morrison A, Choudhary G, Wu W (2019). Home-Based Cardiac Rehabilitation Alone and Hybrid With Center-Based Cardiac Rehabilitation in Heart Failure: A Systematic Review and Meta-Analysis. J Am Heart Assoc.

[12] Ghisi GLDM, Xu Z, Liu X, Mola A, Gallagher R, Babu AS, Yeung C, Marzolini S, Buckley J, Oh P, Contractor A, Grace SL (2021). Impacts of the COVID-19 Pandemic on Cardiac Rehabilitation Delivery around the World. Glob Heart.

[13] Doyle J, Murphy E, Gavin S, Pascale A, Deparis S, Tommasi P, Smith S, Hannigan C, Sillevis SM, van LC, Lastra J, Galvin M, Sheridan P, Tompkins L, Jacobs A, Moreira MM, Medina J, Boyle G, Dinsmore J ProACT - A Digital Platform to Support Self-Management of Multiple Chronic Conditions: Findings in Relation to Engagement during a one-year Proof-of-Concept Trial. JMIR Prepr.

[14] Doyle J, Murphy E, Kuiper J, Smith S, Hannigan C, Jacobs A, Dinsmore J (2019). Managing multimorbidity: identifying design requirements for a digital self-management tool to support older adults with multiple chronic conditions. CHI '19: Proceedings of the 2019 CHI Conference on Human Factors in Computing Systems.

[15] Bellet RN, Adams L, Morris NR (2012). The 6-minute walk test in outpatient cardiac rehabilitation: validity, reliability and responsiveness--a systematic review. Physiotherapy.

[16] American College of Sports Medicine (2017). ACSM’s guidelines for exercise testing and prescription.

[17] Borg G (1998). Borg’s perceived exertion and pain scales.

[18] Irish Association of Cardiac Rehabilitation (2013). Cardiac Rehabilitation Guidelines 2013.

[19] Karvonen MJ, Kentala E, Mustala O (1957). The effects of training on heart rate; a longitudinal study. Ann Med Exp Biol Fenn.

[20] Holland AE, Spruit MA, Troosters T, Puhan MA, Pepin V, Saey D, McCormack MC, Carlin BW, Sciurba FC, Pitta F, Wanger J, MacIntyre N, Kaminsky DA, Culver BH, Revill SM, Hernandes NA, Andrianopoulos V, Camillo CA, Mitchell KE, Lee AL, Hill CJ, Singh SJ (2014). An official European Respiratory Society/American Thoracic Society technical standard: field walking tests in chronic respiratory disease. Eur Respir J.

[21] Ware J, Kosinski M, Keller SD (1996). A 12-Item Short-Form Health Survey: construction of scales and preliminary tests of reliability and validity. Med Care.

[22] Braun V, Clarke V (2006). Using thematic analysis in psychology. Qualitative Research in Psychology.

