# Supplementary material for: Delivering Cardiac Rehabilitation Exercise Virtually Using a Digital Health Platform (ECME-CR): Protocol for a Pilot Trial
Source: JMIR Res Protoc. 2021 Oct 7;10(10):e31855. doi: 10.2196/31855 (PMC8532019; doi:10.2196/31855)
Supplement: Multimedia Appendix 1 [file resprot_v10i10e31855_app1.docx]

# Appendix 1 – Educational Content

## What is Aerobic Exercise?

Aerobic exercise is any type of physical activity that is planned and structured and uses the large muscles in the body. It can include activities like brisk walking, swimming, running, or cycling.
It is also known as cardiovascular exercise or “cardio.”

During aerobic exercise your breathing and heart rate will increase. Aerobic exercise helps keep your heart, lungs, and circulatory system healthy.

Other benefits of aerobic exercise include:

- Improved cardiovascular fitness
- Improved blood pressure, blood sugar levels and cholesterol
- Weight control
- Improved self-esteem and reduced stress and anxiety

### How much aerobic exercise should you be doing?

| Frequency | At least three times per week.Start off by doing a few days a week and you can gradually build up over time. |
| --- | --- |
| Intensity | Moderate – you should be able to complete a sentence but not hold a conversation as you exercise. |
| Time | Aim for 30-60 minutes per session, totalling 150 minutes a week. |
| Type | Walking, swimming, cycling or any type of exercise that involves large muscle groups |

### Remember to warm-up and cool-down

The warm-up and cool-down are vital components of your workout.

**Warm-up**

Minimum of 10-15 minutes. Should feel ‘light’ in intensity. Gradually increase the intensity of exercise to get the heart pumping, to get the muscles warm, and to prepare your body for the main conditioning phase.

**Cool-down**

Don’t skip it! Your cool-down period should be a minimum of 10 minutes. It’s really important that you gradually slow down to avoid developing any symptoms post exercise such as nausea, dizziness or chest pain. Include some stretches at the end of your workout to improve flexibility and help with recovery.

### What else can I do?

Any activity that gets you moving and makes you feel slightly out of breath and with the heart pumping will be beneficial.

Gardening, dancing and even some housework can be considered moderate intensity physical activity.

### How should I feel during my session?

- Listen to your body and think about how it compares to how you feel during your exercise classes.
- Your breathing rate and heart rate should increase as you exercise but you should still be able to talk in a sentence but not hold a full conversation.
- It is fine to feel that you are exerting yourself so long as you are not struggling. If you feel exhausted you may have worked too hard or for too long.
- **If you experience any changes in symptoms such as chest pain, dizziness or worsening breathlessness it is extremely important to stop what you are doing and seek medical advice.**

### Other things to consider

- Having rest days are equally as important as exercise days, giving your body time to recover and repair.
- Make sure you have taken your medications, as prescribed, prior to any exercise.
- Don’t exercise immediately after eating a large meal.
- Remember to stay hydrated – take a bottle of water with you as you exercise.
- Do not exercise if you feel unwell.
- Hot, cold or windy weather will make the exertion more difficult, so reduce the intensity and the amount of time you are exercising for in these conditions.

## Strength Training

Strength Training is also called resistance or weight training. It includes any exercise where you lift a load or use your bodyweight as resistance. The aim is to improve the strength or endurance of your muscles.

### Why do strength training

- As well as improving your muscle strength, strength training helps build strong bones.
- Other benefits of strength training include:
  - Improves blood pressure control
  - Reduces risk of disease
  - Improves well-being and quality of life
  - Helps to maintain a healthy body weight and shape
  - Makes everyday activities easier to perform
  - Reduces the risk of falls, especially in older people.

### How much strength training should you be doing?

| Frequency | 2-3 times per week. No more than four times a week. It is recommended to wait 48 hours between each strength training session. |
| --- | --- |
| Intensity | Start with 1 set of between 10 -15 repetitions (reps) for each muscle group.  To progress, increase the number of sets up to 4 with a minute rest in between  If you cannot perform at least 10 reps one after the other then the weight is too heavy. |

### Equipment

- You can perform strength training using machines or weights if you have access to a gym, resistance bands or your own body weight.
- If you don’t have access to a gym, you can also use items in your home as weights, such as milk cartons, water bottles, or a backpack.
- Always start with a lower weight and build up to heavier weights.
- You want to feel your muscles working, especially by the last couple of reps.

### Tips for strength training

- Always warm-up before participating in any exercise. Before strength training, practice the involved movements with no weights to warm-up the muscles.
- Aim to include exercises that target all the large muscles of the body.
- Move with control and maintain good posture. Take your time through the movement - 2 seconds on the way up and 2 seconds on the way down
- Keep the breath flowing and avoid holding your breath. Try breathing out during the hard part and in during the easy part.
- Avoid over-gripping weights/bands.
- It is normal to feel some muscle soreness up to 48 hours after strength training.
- Cool-down by stretching the muscles used. This will relax the muscles and help improve recovery.

## Stretching and Flexibility

- Flexibility is the ability to move a joint or group of joints and the surrounding tissue through a complete range of motion.
- Flexibility training includes stretching exercise which help lengthen muscles and may include activities like yoga, Tai chi and Pilates.
- Improving your flexibility can help you move more comfortably and efficiently throughout the day and also help aid in recovery after exercise.
- Some areas of your body may be more flexible than others.

### The Benefits

- Relieves muscle tightness and tension
- Improves circulation
- Reduces risk of injury
- Improves posture
- Improves mood and wellbeing
- Relieves stress and promotes relaxation

### How to do flexibility training

You don't have to take a dedicated class or session for flexibility. You can simply add a few minutes of stretching to the end of you daily workout, or take 5-10 minutes to stretch in the morning after you get out of bed.

| **Frequency** | It can take several weeks of consistent, daily stretching to increase flexibility. Stretch after each training session with stretches that will target the largest joints in your body. A dedicated stretching session can also be very beneficial. Aim for 2-3 times per week at a minimum to improve flexibility. |
| --- | --- |
| **Intensity** | Stretch to the point of resistance or mild discomfort. |
| **Time** | Maintain the stretch position for 15-30 seconds to begin. Repeat each stretch three times. |
| **Type** | Stretch each major muscle group, paying particular attention to the muscles that you feel are tight. Stretching should be completed statically, i.e., gradually stretching through a muscle’s full range. Gently release the stretch at the end. |

### Tips for Stretching

- Avoid bouncing, bobbing or jerky motions as you stretch.
- Avoid overstretching – stretch to the point of resistance or mild discomfort. Never push yourself to the point of pain. Begin each stretch slowly and gently.
- To be most beneficial and to avoid injury, stretch when your muscles are warm, i.e. at the end of your workout. Stretching when muscles are cold could lead to a strain or pull.
- Focus on your breath and avoid holding your breath when you are stretching.
- Stretching before an exercise session is generally not recommended.
- Transition slowly between stretches.
