# Supplementary material for: Delivering Cardiac Rehabilitation Exercise Virtually Using a Digital Health Platform (ECME-CR): Protocol for a Pilot Trial
Source: JMIR Res Protoc. 2021 Oct 7;10(10):e31855. doi: 10.2196/31855 (PMC8532019; doi:10.2196/31855)
Supplement: Multimedia Appendix 2 [file resprot_v10i10e31855_app2.docx]

# Appendix 2 – Exercise Program

## Warm-up

15 minutes. Performed seated or standing.

| 1 | Sitting marching feet | Look left and right (1 min) |
| --- | --- | --- |
|  |  | Roll shoulders forward (1 min) |
|  |  | Roll shoulders backwards (1 min) |
|  |  | Marching, arms by side (1 min) |
| 2 | Toe tap in front | Arm curls, 1 arm (1 min) |
|  |  | Raise 1 arm in front (1 min) |
|  |  | Running arms (1 min) |
|  |  | Marching, arms at side (1 min) |
| 3 | Heel Digs | Arm curls, 2 arms together (1 min) |
|  |  | Raise 2 arms in front (1 min) |
|  |  | Running arms (1 min) |
|  |  | Marching, arms at side (1 min) |
| 4 | Toe tap to side | Raise 1 arm to side (1 min) |
|  |  | Breast stroke swim arms (1 min) |
|  |  | Raise arms to side (1 min) |

## Main Circuit

The circuit consists of cardiovascular (CV) exercises and active recovery (AR) exercises. Active recovery exercises are designed to provide rest intervals between cardiovascular work stations.

Program starts with alternating 2 minutes of CV exercise with 12-15 reps on an AR exercise. Duration of CV work is increased over the weeks.

| Level 1 | Week 1-2 | 5 CV and 5 AR alternating stations (10 minutes CV) |
| --- | --- | --- |
| Level 2 | Week 3-4 | 6 CV and 4 AR alternating stations (12 minutes CV) |
| Level 3 | Week 5-6 | 7 CV and 3 AR alternating stations (14 minutes CV) |
| Level 4 | Week 7-8 | 8 CV and 2 AR alternating stations (16 minutes CV) |

| **Examples of CV and AR Exercises** | | | |
| --- | --- | --- | --- |
| 1 | CV | Walking | Up and down hallway or on the spot.  Progress to walking faster, then alternating walking with jogging. |
| 2 | AR | Bicep curl | With free weights. 12-15 repetitions. Keep the feet moving. |
| 3 | CV | Lunge | Forward or backward lunge. Progress by increasing range of movement and/or adding bigger arm movements. |
| 4 | AR | Wall Press | 12-15 repetitions. Progress by taking the body further away from the wall and/or doing the exercise more slowly. |
| 5 | CV | Side taps | Progress by increasing range of movement and/or adding graduated arm movements |
| 6 | AR | Upright row | With free weights. 12-15 repetitions. Keep the feet moving. |
| 7 | CV | Step up | Step up and down on the bottom step of stairs or step in house. Progress by climbing up and down stairs. |
| 8 | AR | Seated low row | With free weights. 12-15 repetitions. Keep the feet moving. |
| 9 | CV | Hamstring curls | Progress by increasing range of movement and/or adding graduated arm movements. |
| 10 | AR | Lateral Raise | With free weights. 12-15 repetitions. Keep the feet moving. |
| 11 | CV | Sit-stand | Using a sturdy chair, sit down and stand up. Take a break if needed but keep feet moving. |
| 12 | AR | Frontal Raise | With free weights. 12-15 repetitions. Keep the feet moving. |
| 13 | CV | Knee Raise | Progress by increasing range of movement and/or adding graduated arm movements |
| 14 | AR | Tricep kickback | With free weights. 12-15 repetitions. Keep the feet moving. |

## Cool-down

10 minutes. Performed seated or standing.

| 1 | Toe taps to side (2 mins) |
| --- | --- |
| 2 | Heel digs (2 mins) |
| 3 | Slide toe tap in front (1 min) |
| 4 | Heel Raises (1 min) |
| 5 | Upper back stretch (30 sec) |
| 6 | Chest stretch (30 sec) |
| 7 | Hamstring stretch (30 sec each side) |
| 8 | Deep breathing exercise (2 mins) |

## Bibliography

[1]. The British Association for Cardiovascular Prevention and Rehabilitation (2018). Physical Activity and Exercise in the Management of Cardiovascular Disease.
